# Primary Retroperitoneal Smooth Muscle Tumor of Uncertain Malignant Potential (STUMP): A Diagnostic Enigma

**DOI:** 10.7759/cureus.33332

**Published:** 2023-01-03

**Authors:** Chinniahnapalya Pandurangaiah Hariprasad, Anil Kumar, Gupta Rohit, Surabhi Surabhi, Deepti Bhatt

**Affiliations:** 1 General Surgery, All India Institute of Medical Sciences, Patna, IND; 2 Surgery, All India Institute of Medical Sciences, Patna, IND; 3 Pathology and Laboratory Medicine, All India Institute of Medical Sciences, Patna, IND

**Keywords:** smooth muscle tumors of uncertain malignant potential, retroperitoneal sarcoma, leiomyosarcomas, leiomyomas, soft tissue tumor

## Abstract

Retroperitoneal sarcomas represent a group of rare malignant neoplasms with complex clinical management and often a poor prognosis. An elderly male presented with a slowly progressive, right-sided abdominal lump for four months associated with loss of appetite and abdominal discomfort. Abdominal examination revealed an apparent retroperitoneal lump in the right lumbar and umbilical region, which was well-defined, and firm in consistency with the bosselated surface. Contrast-enhanced computed tomography (CECT) of the abdomen and pelvis revealed a heterogenous lobulated malignant appearing retroperitoneal lesion arising from the right anterior perirenal space with a differential of retroperitoneal sarcoma. Wide local excision of the tumor was done. Histopathology of the lesion revealed a smooth muscle tumor of uncertain malignant potential (STUMP). The patient is asymptomatic and recurrence-free after 24 months of follow-up.

## Introduction

Retroperitoneal sarcomas represent a group of rare malignant neoplasms, complex clinical management, and often a poor prognosis with about 70 different histological variants and an annual incidence of 0.76 new cases per 100,000 [1]. Smooth muscle tumors of uncertain malignant potential (STUMP) are a rare sub-group of heterogenous tumors that are predominantly uterine in origin. Histologically, it is a part of the spectrum of deep soft tissue tumors, the two ends of which are leiomyomas and leiomyosarcomas [2]. Rare reports in unusual locations such as retroperitoneum and clinical presentations ranging from benign mass to metastatic pulmonary nodules make STUMP a medical enigma necessitating relentless reporting of any unique representations of the same [3]. This research has been reported in line with Surgical CAse REport (SCARE) criteria and compliant with Preferred Reporting Of CasE Series in Surgery (PROCESS) guidelines [4,5]. This article was previously presented as a poster at the 81st Association of Surgeons of India Annual Conference (ASICON) on December 18, 2021.

## Case presentation

**Figure 1:**
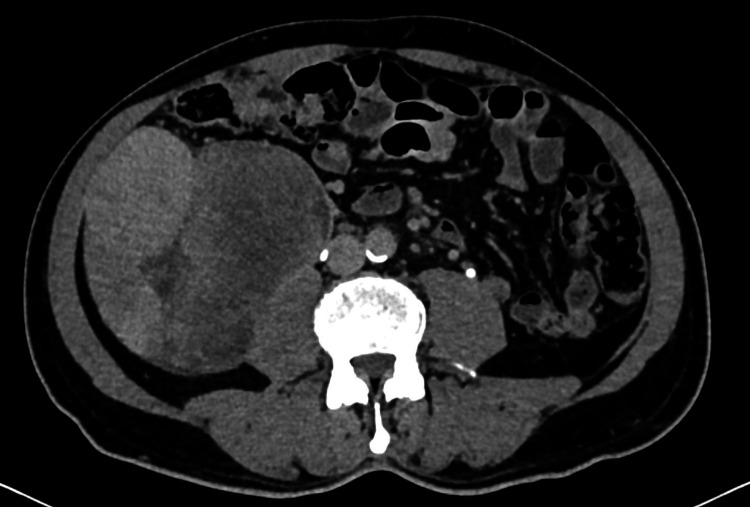
Computed tomography scan, axial section showing a huge mass in the right anterior pararenal space, abutting inferior vena cava

A well-built gentleman in his 60s, without any comorbidity, presented to the general surgery outpatient department with a painless, non-progressive lump in the right side of the abdomen, noticed four months ago, without a history of fever, vomiting, and abdominal distension. On examination, the size of the lump was 11 x 15 cm in the right lumbar and umbilical region, located in the retroperitoneum, which was apparent on inspection, with ill-defined margins, firm consistency, and bosselated surface on palpation. General physical examination was unremarkable with no inguinal or cervical lymphadenopathy. Ultrasonography (USG) of the abdomen and pelvis was done as an adjunct to clinical examination suggesting a heteroechoic mass of size 10 x 15 cm, in the retroperitoneum. Contrast-enhanced computed tomography (CECT) of the abdomen revealed a well-defined, lobulated heterogeneous lesion of size 11.6 x 11.2 x 15 cm in the retroperitoneum and right anterior perirenal space. Inferolateraly, the lesion was seen infiltrating the right psoas muscle with focal loss of the fat plane. Laterally, the lesion is seen abutting the inferior vena cava (IVC), right ureter, and adjacent bowel loops without infiltration. No fat or calcification was noted within the lesion (Figure 1).

**Figure 2:**
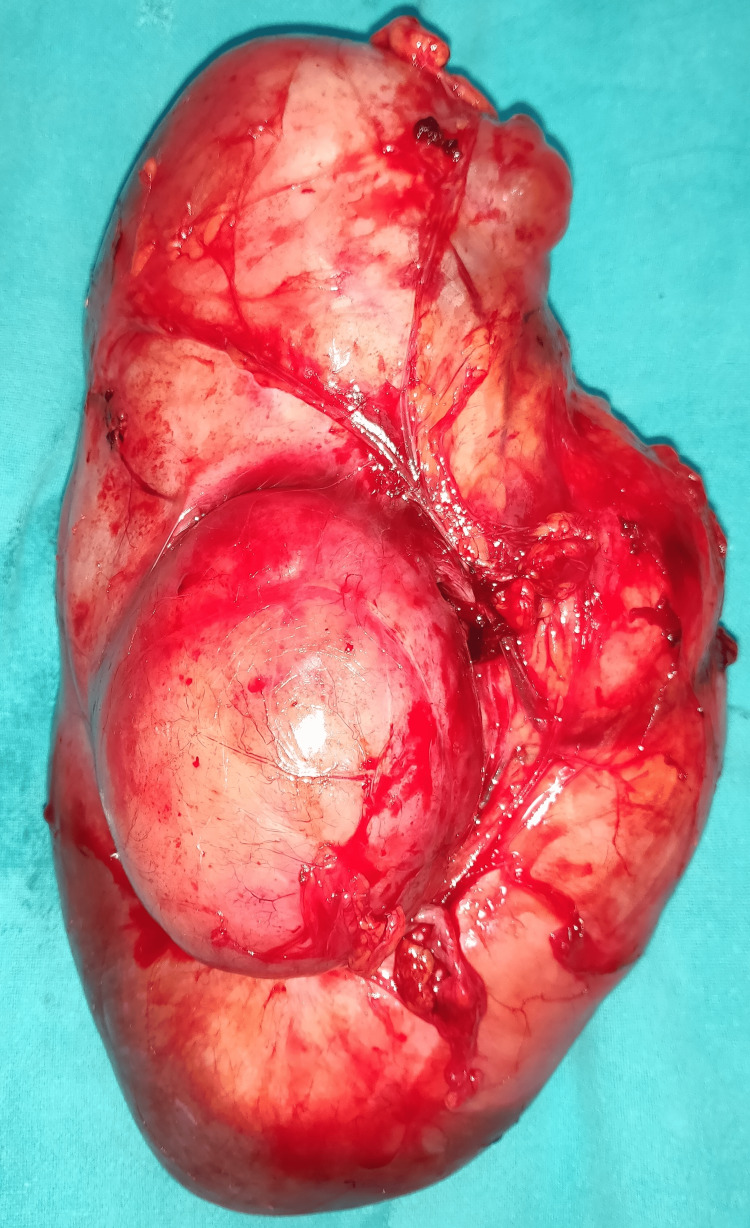
Postoperative specimen showing the lobulated retroperitoneal mass with congested blood vessels

No liver nodules were noted. CECT of the thorax showed no evidence of lung metastasis. Based on the CECT findings, a provisional diagnosis of retroperitoneal sarcoma was made and after a detailed review with the tumor board comprising specialists from radiodiagnosis, surgical oncology, and radiation oncology, surgical intervention was planned. Wide local excision of the tumor with 5 centimeters of margin was performed, and the negative margins were confirmed by intraoperative frozen section. There were no intraoperative and immediate postoperative complications and the wound was healthy. After an uneventful postoperative course of five days, the patient was discharged. Macroscopic examination revealed a large (24 x 13 x 10 cm) mass. The outer surface of the mass was smooth and well-encapsulated with congested blood vessels (Figure 2).

**Figure 3:**
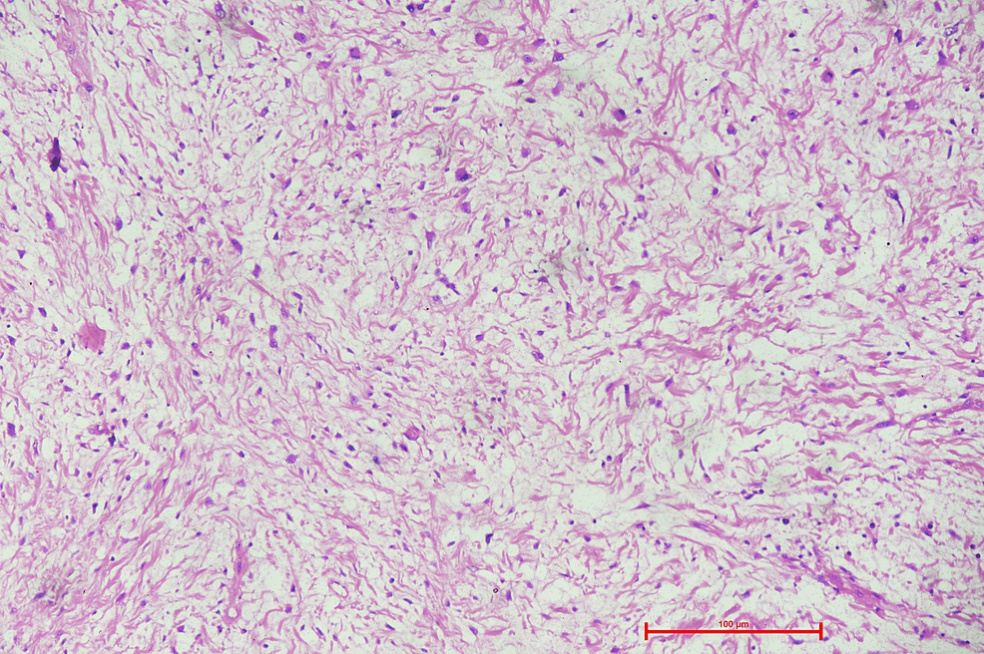
Photomicrograph showing the presence of spindle cells displaying moderate pleomorphism in the form of vesicular chromatin, prominent nucleoli, and a few interspersed multinucleate giant cells (Hematoxylin and Eosin 100x resolution)

**Figure 4:**
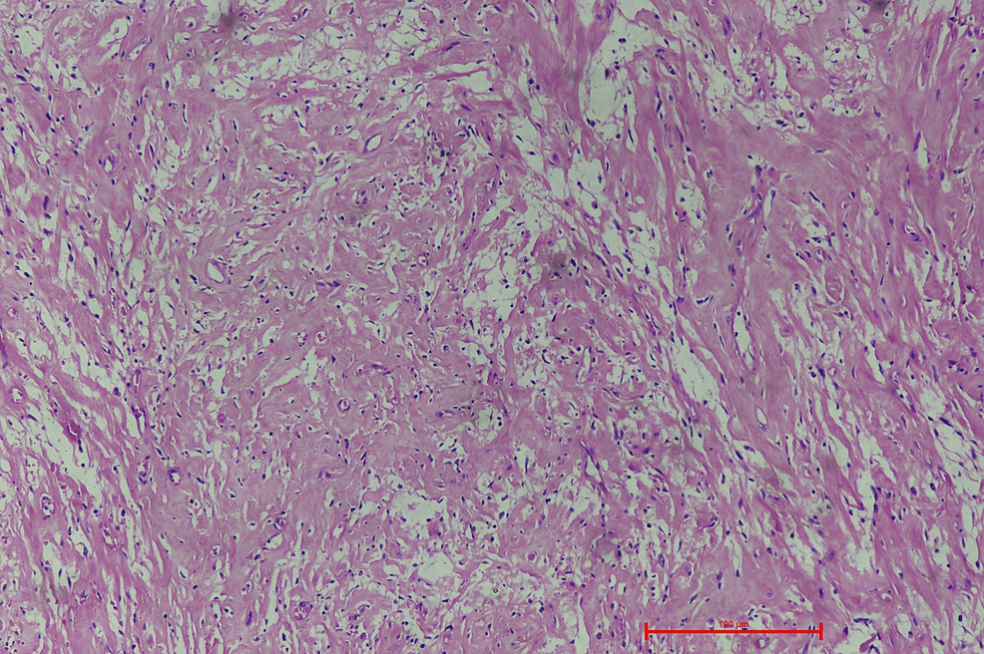
Photomicrograph showing extensive collagenization (Hematoxylin and Eosin 100x resolution)

The cut surface was bosselated, nodular, solid homogenous, greyish-white mass with myxoid and cystic areas. The largest cyst measured 5 x 6 x 4 cm, which yields straw-colored fluid. The findings were consistent with the CECT done preoperatively. The postoperative microscopic findings included tumor mass of variable cellularity with spindle cells arranged in a storiform pattern. These spindle cells are large, with a severe pleomorphic nucleus, vesicular chromatin, prominent nucleoli, and a moderate amount of cytoplasm, in an abundant collagenous stroma (Figure 3 and Figure 4).

**Figure 5:**
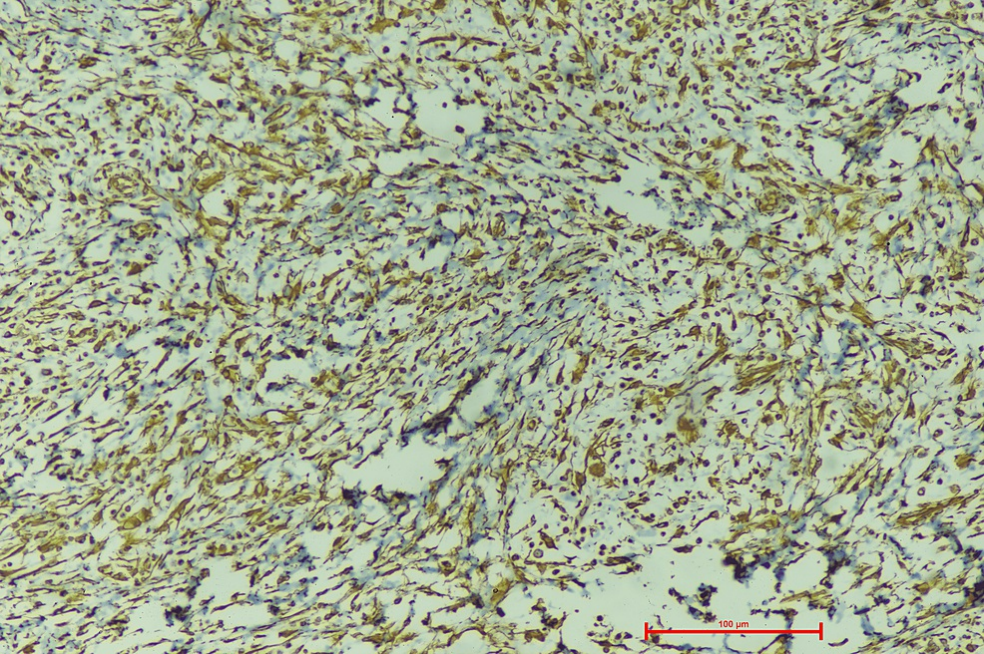
Vimentin stained section showing membranous and cytoplasmic positivity in tumor cells

Upon vimentin stain, nuclear and cytoplasmic positivity was seen, suggestive of mesenchymal origin (Figure 5).

H-caldesmon stain was also positive, narrowing the diagnosis down to smooth muscle tissue. Tumor cells were also diffusely positive for smooth muscle actin and desmin, PanCK, S-100, WT-1, and ALK-negative. There were areas of necrosis corresponding to less than 50% of tumors noted. Mitotic count was 4-5 figures/high power field. Margins were free of tumors. The patient is in regular follow-up at an interval of every six months and was last reviewed after 24 months of surgery, and he was asymptomatic without recurrence which was confirmed after clinical and radiological investigations. The multidisciplinary tumor board discussion suggested no role of adjuvant therapy at present but to keep the patient in close follow-up.

## Discussion

Primary retroperitoneal neoplasms are a rare, heterogenous group of tumors not arising from any retroperitoneal organ [6]. These lesions are difficult to diagnose due to overlaps in imaging features and clinical presentations. Smooth muscle tumors comprise leiomyomas and leiomyosarcomas, of which the latter form a significant portion of retroperitoneal sarcomas. These present in the fifth and sixth decades with female preponderance, as large well-circumscribed masses on CT [6]. Leiomyomas, however, are extremely rare in retroperitoneum, therefore is a need for a strict criterion to establish the benign nature of a deep soft tissue tumor in such a location. Histologically, leiomyosarcoma, as reported by Bell et al. [6], should include at least two of the following criteria: diffuse moderate-to-severe atypia, a mitotic count of at least 10 mitotic figures per 10 high power field (HPF) and tumor cell necrosis. Atypia, even of a focal nature warrants evaluation of the mitotic activity. In retroperitoneal location, the mitotic activity of <5/HPF suggests benign in nature and while higher counts than this might still be benign, due to rare presentations, these lesions, known as STUMP, remain in a grey zone between the two aforementioned categories [3,7]. The classification systems and management protocols of STUMP are therefore developed in accordance with gynecological tumors. We report a rare case of a male patient in his 60s presenting with a retroperitoneal mass, which on pathological examination shows spindle cells, with focal necrosis, myxoid changes, and a mitotic count of 4-5/HPF. Primary retroperitoneal STUMP is an extremely rare entity. Genomic profiling studies to identify the similarities and differences of STUMP with leiomyomas and leiomyosarcoma are being carried out to decrease the diagnosis of STUMP [8]. Retroperitoneal STUMP without antecedent pathology has rarely been reported in the literature and many of the STUMP reported are of uterine origin [9-11]. Surgery has promised to be the potentially curative treatment in primary retroperitoneal tumors. Neoadjuvant therapies like radiotherapy have been tried in a large multicentric trial without any encouraging results [12]. The multidisciplinary approach to a case of retroperitoneal sarcoma would lead to better outcomes and strict adherence to the follow-up protocol can help in early diagnosis of recurrence and effective management of the same.

## Conclusions

A retroperitoneal sarcoma is a rare group of soft tissue sarcoma with varied presentation and complex histological categories associated with poor prognosis. Soft tissue tumor of uncertain malignant potential is even rare entity that can neither be categorized as benign nor as a malignant entity. Surgery after multidisciplinary tumor board discussion and strict follow-up adherence can have good promising outcomes.
